# Getting back to work after injury: the UK Burden of Injury multicentre longitudinal study

**DOI:** 10.1186/1471-2458-12-584

**Published:** 2012-08-01

**Authors:** Denise Kendrick, Yana Vinogradova, Carol Coupland, Nicola Christie, Ronan A Lyons, Elizabeth L Towner

**Affiliations:** 1 Division of Primary Care, University Park, Floor 13 Tower Building, Nottingham, NG7 2RD, UK; 2 Centre for Transport Studies, Department of Civil, Environmental and Geomatic Engineering, UCL Gower Street, London, WC1E 6BT, UK; 3 College of Medicine, Swansea University, Grove Building, Singleton Park, SA2 8PP, Swansea; 4 Centre for Child & Adolescent Health, University of the West of England, Oakfield House, Oakfield Grove, Clifton, Bristo, l BS8 2BN, UK

## Abstract

**Background:**

Injuries to working age adults are common and place a considerable burden on health services accounting for more than 10% of GP sick notes and 14% of those claiming benefits because they are unable to work in the UK. General practitioners (GPs) currently assess fitness to work and provide care and referral to other services to facilitate return to work (RTW). Recent UK recommendations suggest replacing GP sickness certification with independent assessments of fitness to work after four weeks sick leave. The impact of a wide range of injuries on RTW and subsequent need for independent fitness to work assessments has not been well studied in the UK. The aim of this study was to quantify RTW and factors predicting RTW following a wide range of injuries.

**Methods:**

We used a multicentre longitudinal study, set in four acute NHS Trusts in the UK which recruited emergency department (ED) attenders and hospital admissions for injury and included those aged 16–65years that were employed or self-employed before the injury. Participants were followed up by postal questionnaire at 1, 4 and 12 months post injury to measure health status (EQ-5D), recovery, use of health and social services, time off work in the preceding month and work problems amongst those who had RTW. Multivariable Poisson regression with a robust variance estimator was used to estimate relative risks for factors associated with RTW.

**Results:**

One month after injury 35% of ED attenders had fully RTW. The self employed were more likely (RR 1.70, 95% CI 1.17 to 2.47 compared with employed) and the moderate/severely injured less likely to RTW (RR 0.48, 95% CI 0.32 to 0.72 compared with minor injuries). At four months, 83% of ED attenders had RTW and self employment and injury severity remained significant predictors of RTW (self employment RR 1.15, 95% CI 1.03 to 1.30; moderate/severe injury RR 0.79, 95% CI 0.68 to 0.92). At four months 57% of hospital admissions had RTW. Men were more likely than women to RTW (RR 1.94, 95% CI 1.34 to 2.82), whilst those injured at work (RR 0.49, 95% CI 0.27 to 0.87 compared with at home) and those living in deprived areas (most deprived tertile RR 0.59, 95% CI 0.40 to 0.85 and middle tertile RR 0.61, 95% CI 0.40 to 0.93) were less likely to RTW. Health status was significantly poorer at one and four months after injury than before the injury and was significantly poorer amongst those that had not RTW compared to those that had. Problems with pain control, undertaking usual activities, mobility and anxiety and depression were common and persisted in a considerable proportion of participants up to four months post injury.

**Conclusions:**

Injuries have a large impact on time off work, including amongst those whose injuries did not warrant hospital admission. The majority of injured people would require an in-depth fitness for work assessment if recent UK recommendations are implemented. Many people will have on-going pain, mobility problems, anxiety and depression at the point of assessment and it is important that patients are encouraged to use primary care services to address these problems. A range of factors may be useful for identifying those at risk of a slower recovery and a delayed RTW so that appropriate interventions can be provided to this group.

## Background

Injuries to working age adults are a major public health problem, resulting in more than 400,000 hospital admissions in England [1] and approximately 2.8 million emergency department (ED) attendances in the UK [2] annually. There is also evidence that those who have suffered injury have greater health service use in the years following their injury than the general population, and in some cases this can continue for many years following the injury [3].

Injuries also have a major impact on work absence in the UK. More than 10% of all general practitioner (GP) sick notes are issued for injuries [4] and they are the second most common reason for Employment Support Allowance (the benefit paid in the UK for incapacity) accounting for 14% of all claimants [5].

As there is evidence that work has a positive impact on health, [5] helping people return to work (RTW) following illness or injury is central to the UK Governments strategy for the health and well being of working age adults [6]. Sickness certification in the UK is currently provided by GPs and a new “fit-note” was recently introduced to enable GPs to make recommendations to employers to facilitate RTW, accompanied by a national training programme for GPs [7]. A more recent independent review now recommends major changes to the certification of sickness absence in the UK, removing the GP from this task once sickness absence has lasted four weeks. It proposes an independent service to provide an in-depth assessment of physical and mental function and to advise about supporting people in RTW [5]. This is likely to have major implications for those suffering injuries and for GPs who provide continuing care, on-going support and co-ordination of other services for people after injury.

Although there is a body of literature exploring the impact of injuries on work in other countries, this may not be generalisable to the UK because the types of occupations, compensation systems, health care and welfare systems vary between countries. Little research to date has examined the factors associated with RTW following a wide range of injuries in the UK. A recent systematic review of prognostic factors for RTW after orthopaedic trauma found 15 longitudinal studies, only two of which were from the UK [8]. The first showed that RTW was more strongly associated with the amount of time a person had been off work, psychological problems and age than with clinical characteristics of the injury [9]. The second found that perceived blame, litigation and disrupted social activities were more important than injury severity in predicting RTW at six months and that post traumatic stress disorder avoidance symptoms and disrupted usual physical activities were the only factors associated with RTW 18 months post injury [10]. The analyses in this paper have been undertaken to quantify the impact of a wide range of injuries on RTW, health care use and health status in the UK and explore factors associated with RTW via a secondary analysis of data from the UK Burden of Injury Study (UKBOI) [11].

## Methods

### Study design

The UKBOI study was a prospective longitudinal study set in four acute NHS Trusts in the UK. The analyses presented in this paper use a subset of participants from the UKBOI study.

### Participants

The four study centres participating in the UKBOI study were Swansea, Nottingham, Bristol and Guildford. These cities and towns had populations in 2006 ranging from 132,000 in Guildford to 414,000 in Bristol, with working age adults comprising between 63% (Swansea) and 69% (Nottingham) of the population [12]. Employment rates in 2006 ranged from 64% in Nottingham to 82% in Guildford [13]. The 2001 census found the percentage of the population in higher and intermediate managerial, administrative or professional occupations ranged from 16% in Nottingham to 34% in Guildford and the percentage on state benefits, unemployed or in the lowest grade occupations ranged from 12% in Guildford to 21% in Nottingham [14]. Age standardized mortality rates from unintentional injuries between 2008 and 2010 varied between the study centres, being significantly higher in Nottingham and lower in Guildford than those for England [15]. Directly comparable rates are not available for Swansea, but the age standardised unintentional injury mortality rate in 2003 was significantly higher in Wales than in England, and similar to that for the East Midlands region which contains Nottingham [16].

Participants for the UKBOI study were people aged 5 years and over attending EDs or admitted to hospital in one of the four study centres with a wide range of injuries, including fractures/dislocations, lacerations, bruises/abrasions, sprains, burns/scalds, and head, eye, thorax and abdominal injuries [11]. In the UK, trauma cases usually attend EDs, with those that are assessed in the ED as needing admission being admitted to hospital. ED attenders were therefore defined as those attending ED who were not admitted to hospital. Hospital admissions were defined as those admitted to hospital, whether or not they attended ED immediately prior to their admission. Injuries had to have occurred within 2 weeks of attendance for ED treated patients and four weeks for hospital admitted patients. Patients had to be able to give consent and complete questionnaires or to have a suitable proxy who could assent to their inclusion and agree to complete future questionnaires. Those without permanent UK addresses were excluded. Participants were recruited face-to-face during their hospital admission or attendance between September 2005 and April 2007. The analyses presented in this paper were restricted to those aged 16–65 years inclusive that were employed or self-employed before the injury.

### Data collection

Participants self-completed questionnaires at recruitment and by post at 1, 4 and 12 months post injury. Once participants reported complete recovery (defined as the injury no longer affecting them in any way), no further questionnaires were sent. Questionnaires were designed specifically for this study. They included an open question about how the injury happened, plus closed questions asking about activity at time of injury, place of injury, whether a motor vehicle was involved and injury intent. Socio-demographic data included closed questions, taken from the 2001 UK Census on accommodation type, number of rooms in household, number of people living in household, number of cars or vans by use of household members and ethnic group [17]. The participant’s postcode was obtained from direct enquiry at recruitment, and from this, the Townsend deprivation score was obtained. The Townsend deprivation score is a composite deprivation index comprising four indicators: unemployment, housing tenure, car ownership and overcrowding [18]. Information on whether the participant had any disability or long term health problems prior to the injury which limited usual activity was ascertained using a dichotomous question. General health prior to injury was measured using the EQ5D index and Visual Analogue Scale (VAS) [19]. Employment status prior to injury was ascertained by asking if participants were employed, self-employed or not working. Self-completed follow-up questionnaires collected data on general health (EQ-5D and VAS) and recovery using a dichotomous question (defined as whether the injury still affected them in any way on the day of completing the questionnaire). Data on RTW was collected by asking the number of days that participants did not attend work due to their injury in the preceding four weeks. Data on use of health and social services was collected by asking participants to specify the number of days on which they used a range of pre-specified services in the preceding four weeks because of their injury. Work problems for those who had RTW were measured using the Work Limitations Questionnaire [20]. Data on the body part injured and injury severity was ascertained from medical records and injury severity was scored using the Abbreviated Injury Scale (AIS) [21].

We defined full RTW as not being prevented from working for any days in the last four weeks. Where participants did not answer this question but reported they had returned to normal activities on other questions, we assumed full RTW. Those reporting they had fully recovered from their injury at one month (100 participants) were not sent a four month questionnaire; hence we assumed they had fully RTW at four months.

### Statistical analysis

Twelve month data were not analysed as only 20 participants had not RTW at this point. We ran separate analyses for ED attenders and hospital admissions, as previous studies suggest RTW may vary by admission status [22]. Only 10 participants who had been admitted to hospital had RTW one month following injury, so prognostic factors for RTW were only explored for those admitted to hospital using the four month data. Outcomes were commonly reported, hence Poisson regression, with a robust variance estimator [23,24] was used to estimate the relative risk of RTW at one and four months post injury. Age and deprivation were categorised as each had a non-linear relationship with RTW. Injury type was categorised into upper limb, lower limb and other injuries as numbers were small for some injury types and previous studies suggest prognostic factors may differ between upper and lower limb injuries [8]. Injury severity was categorised into minor (AIS=1) and moderate/severe (AIS=2 or 3). We present two multivariable models for each analysis. The first includes all baseline variables (study centre, age, sex, deprivation, employment status, long-term illness, place of injury, type of injury, severity and injury intent) and the second uses backward stepwise regression, forcing study centre into the model, and retaining other variables if the Wald test for their removal was significant (p<0.05). Observations with missing data were excluded, model assumptions were checked and sensitivity analyses undertaken excluding observations with standardised residual values >2 or<−2 and highly influential observations.

Health status (EQ5D index and VAS scores) was compared between pre-injury and post injury at one and four months post injury using the Wilcoxon matched pairs test. Health status was compared between those who had and who had not RTW at one and four months post injury using the Mann–Whitney U test.

### Ethical approval

Ethical approval was provided by Dyfed Powys Local Ethics Committee (Number: 05/WMW01/23).

## Results

**Figure 1:**
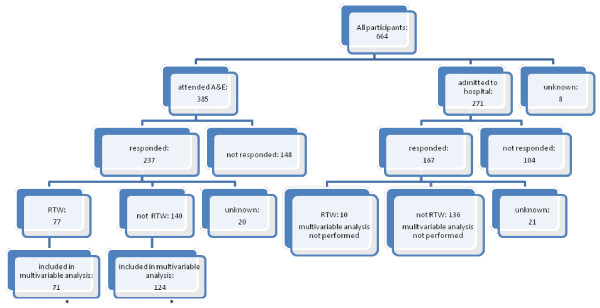
**Responders and non-responders to one month follow up questionnaire and return to work (RTW).** *6 who had RTW and 16 who had not RTW excluded from multivariable analysisdue to missing data on returned questionnaire.

**Figure 2:**
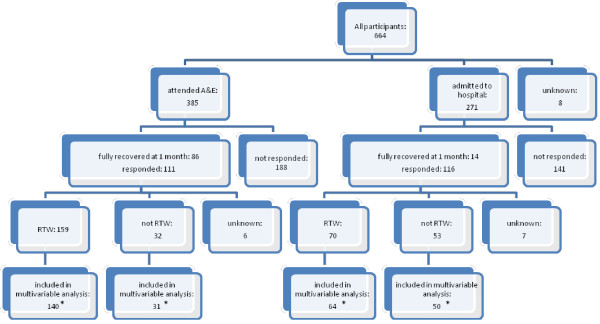
**Responders and non-responders to 4 month follow up questionnaire and RTW.** 19 A&E attenders who had RTW and 1 who had not RTW excluded from multivariable analysis due to missing data on returned questionnaire. *6 hospital admissions who had RTW and 3 who had not RTW excluded from multivariable analysis due to missing data on returned questionnaire.

At baseline 664 participants were included, of these 83% were employed (n=554) and 17% were self employed (n=110). Fifty eight percent (n=385) were ED attenders and 41% (n=271) were hospital admissions. At one month, 62% of both ED attenders and hospital admissions returned questionnaires. Four month data were available on 51% (197/385) of ED attenders and 48% (130/271) of admissions. These figures include those fully recovered at one month who were not sent a four month questionnaire and who were assumed to be fully RTW at 4 months. Data from 51% of ED attenders (195/385) was included in the multivariable analysis at one month and from 44% (171/385) of ED attenders and 42% (114/271) of admissions at four months. (Figures 1 and 2).

At one month, responders were older than non-responders (median 41 years vs. 32 years, p<0.001), more likely to be female (44% vs. 28%, p<0.001), less likely to live in deprived areas (27% in most deprived tertile vs. 39%, 31% vs. 32% in middle tertile and 37% vs. 23% in least deprived tertile, p<0.001), had more severe injuries (60% had moderate or severe injuries vs. 46%, p<0.001) and were less likely to have had their injury at work, on the road or at a sports ground (home 24% vs 23%, work 18% vs. 20%, road 21% vs. 24%, sports ground 13% vs. 18% and other location 25% vs. 12%, p=0.002). At four months, responders were older than non-responders (median 45 years vs. 33 years, p<0.001), more likely to be female (48% vs. 29%, p<0.0001), less likely to live in deprived areas (28% in most deprived tertile vs. 37%, 32% vs. 32% in middle tertile and 37% vs. 26% in least deprived tertile, p=0.02), had more severe injuries (73% had moderate or severe injuries vs. 51%, p<0.001), were more likely to have upper or lower limb injuries and less likely to have other injuries than non-responders (upper limb injuries 25% vs. 22%, lower limb injuries 45% vs. 38%, other injuries 30% vs. 41%, p=0.03) and had a similar pattern in terms of injury location as at one month (home 22% vs. 22%, work 16% vs. 19%, road 22% vs. 25%, sports ground 14% vs. 17%, other location 26% vs. 15%, p=0.045).

### Baseline characteristics

Table 1 shows that at baseline just over half (56%) of ED attenders were male, and almost half (48%) were aged 16–35 years. Most injuries in ED attenders occurred at home (25%), at work (20%) or on the road (19%). Superficial injuries (24%), upper limb fractures (18%) and other lower limb injuries (17%) were the most common injuries. Two fifths (41%) were moderate or severe injuries.

**Table 1:** Baseline characteristics of study participants and their injuries (column percentage)

	**Attended ED (N=385)**		**Admitted to hospital (N=271)**	
	**N (%)**		**N (%)**	
Study centre				
Swansea	87 (22.6)		56 (20.7)	
Bristol	93 (24.2)		56 (20.7)	
Nottingham	126 (32.7)		128 (47.2)	
Surrey	79 (20.5)		31 (11.4)	
Sex:male	216 (56.1)		193 (71.2)	
Age (median, IQR)	36 (27–48)		39 (27–50)	
16 to 35 years	186 (48.3)		115 (42.4)	
36 to 55 years	147 (38.2)		115 (42.4)	
56 to 65 years	52 (13.5)		41 (15.1)	
Ethnicity: White UK	356 (94.7)		254 (95.5)	
Deprivation ( Townsend score tertiles)		[21]		[10]
1 (least deprived)	127 (34.9)		81 (31.0)	
2	120 (33.0)		87 (33.3)	
3 (most deprived)	117 (32.1)		93 (35.6)	
Employment:				
Paid employment	333 (86.5)		215 (79.3)	
Self-employed	52 (13.5)		56 (20.7)	
Long-term illness: None	347 (90.6)	[2]	251 (92.6)	
Place of injury		[6]		[1]
Home	95 (25.1)		60 (22.2)	
Work	76 (20.1)		44 (16.3)	
Road	73 (19.3)		72 (26.7)	
Sports grounds	55 (14.5)		44 (16.3)	
Other	80 (21.1)		50 (18.5)	
Type of injury				
Skull-brain injury	1 (0.3)		9 (3.3)	
Facial fracture, eye injury	18 (4.7)		11 (4.1)	
Spine, vertebrae	18 (4.7)		9 (3.3)	
Internal organ injury	0 (0.0)		3 (1.1)	
Upper extremity fracture	68 (17.7)		37 (13.7)	
Upper extremity, other injury	35 (9.1)		9 (3.3)	
Hip fracture	0 (0.0)		5 (1.8)	
Lower extremity, fracture	57 (14.8)		102 (37.6)	
Lower extremity, other injury	64 (16.6)		16 (5.9)	
Superficial injury, open wounds	94 (24.4)		35 (12.9)	
Burns	13 (3.4)		19 (7.0)	
Other injury	17 (4.4)		16 (5.9)	
Severity				
Minor	226 (58.7)		69 (25.5)	
Moderate or severe	159 (41.3)		202 (74.5)	
Injury intent: Unintentional	359 (96.2)	[12]	237 (89.8)	[7]
EQ5Dmoderate/serious problems				
Mobility	10 (2.6)	[3]	11 (4.1)	
Self-care	1 (0.3)	[3]	3 (1.1)	[1]
Usual activities	10 (2.6)	[4]	7 (2.6)	
Pain/Discomfort	47 (12.3)	[2]	29 (10.7)	[1]
Anxiety/Depression	24 (6.3)	[3]	20 (7.4)	[1]
EQ5D Index	1.00 (1.00-1.00)	[5]	1.00 (1.00-1.00)	[2]
EQ5D VAS	90 (80–97)	[5]	90 (80–99)	

[ ] missing values.

More than two thirds of those admitted to hospital were male (71%), and most were aged 16–35 or 36–55 years (42% each). Most injuries occurred on the road (27%) and at home (22%). Fractures were the most common injury (38% lower and 14% upper limb). Three quarters (75%) were moderate or severe injuries.

### Use of health services and time off work

Table 2 shows that at one month one third of ED attenders (33%) had consulted their GP and 19% had consulted the practice nurse because of their injury. Two thirds (65%) had taken time off work in the previous month, with one third (31%) taking 11 or more days off. Almost half (47%) had taken time off for health care visits and 27% had relatives or friends who had taken time off to care for them. Injuries needing hospital admission had a greater impact on health care use and on work. More than half (58%) had consulted their GP, 81% took 11 or more days off work, 59% took time off for health care visits and 58% had others taking time off to care for them.

**Table 2:** Use of health care services and impact of injury on work in the preceding 28 days at 1 month and 4 months post injury (column percentage)

	**1 month (%)**				**4 months (%)**			
	**Attended ED (N=237)**		**Admission (N=167)**		**Attended ED (N=111)**		**Admission (N=116)**	
Median (IQR) days since injury	38 (34–44)	[40]	36 (32–42)	[10]	161 (135–177)	[9]	154 (131–168)	[7]
Use of health services due to injury								
Consulted GP	73 (32.9)	[15]	93 (58.1)	[7]	23 (20.7)	[0]	41 (35.7)	[1]
Consulted practice nurse	41 (18.6)	[16]	26 (16.5)	[9]	1 (0.9)	[2]	1 (0.9)	[3]
Been to A&E	89 (39.4)	[11]	117 (72.7)	[6]	17 (15.5)	[1]	45 (39.1)	[1]
Hospital Outpatient department	10 (4.5)	[17]	34 (21.8)	[11]	4 (3.7)	[3]	4 (3.6)	[4]
Hospital In-patient	10 (4.7)	[22]	16 (10.5)	[15]	1 (0.9)	[3]	6 (5.4)	[4]
Days with restricted activities		[9]		[10]		[7]		[8]
None	27 (11.8)		2 (1.3)		54 (51.9)		44 (40.7)	
1 to 7 days restricted	58 (25.4)		9 (5.7)		14 (13.5)		8 (7.4)	
8 to 14 days restricted	40 (17.5)		15 (9.6)		5 (4.8)		2 (1.9)	
15 to 28 days restricted	103 (45.2)		131 (83.4)		31 (29.8)		54 (50.0)	
Days off work due to injury		[20]		[21]		[6]		[7]
None	77 (35.5)		10 (6.8)		73 (69.5)		56 (51.4)	
1 to 5 days off work	49 (22.6)		8 (5.5)		9 (8.6)		4 (3.7)	
6 to 10 days off work	24 (11.1)		10 (6.8)		3 (2.9)		4 (3.7)	
11 to 15 off work	10 (4.6)		10 (6.8)		2 (1.9)		6 (5.5)	
16 to 20 days off work	9 (4.1)		8 (5.5)		2 (1.9)		2 (1.8)	
More than 20 days off work	48 (22.1)		100 (68.5)		16 (15.2)		37 (33.9)	
Time off for health care visits		[39]		[58]		[11]		[22]
None	106 (53.5)		45 (41.3)		68 (68.0)		67 (71.3)	
1 to 5 days off work	71 (35.9)		30 (27.5)		21 (21.0)		22 (23.4)	
6 to 10 days off work	5 (2.5)		8 (7.3)		4 (4.0)		1 (1.1)	
11 to 15 off work	5 (2.5)		1 (0.9)		0 (0.0)		1 (1.1)	
16 to 20 days off work	4 (2.0)		0 (0.0)		0 (0.0)		0 (0.0)	
More than 20 days off work	7 (3.5)		25 (22.9)		6 (6.0)		3 (3.2)	
Relatives or friends' time off to care		[26]		[24]		[10]		[10]
None	155 (73.5)		60 (42.0)		94 (93.1)		84 (79.2)	
1 to 5 days off work	45 (21.3)		55 (38.5)		5 (5.0)		14 (13.2)	
6 to 10 days off work	5 (2.4)		16 (11.2)		0 (0.0)		6 (5.7)	
11 to 15 off work	2 (0.9)		5 (3.5)		1 (1.0)		0 (0.0)	
16 to 20 days off work	1 (0.5)		2 (1.4)		0 (0.0)		0 (0.0)	
More than 20 days off work	3 (1.4)		5 (3.5)		1 (1.0)		2 (1.9)	

[ ] missing values.

At four months 21% of ED attenders had consulted their GP because of their injury in the last month, 31% had taken time off work, with 19% taking 11 or more days off, 21% had taken time off for health care visits and 7% had others who had taken time off to care for them. For hospital admissions, 36% had consulted their GP, and 41% took 11 or more days off work, 29% took time off for health care visits and 21% had others taking time off to care for them.

### ED attenders and work status following injury

One month after injury only 35% (77/217) of ED attenders had fully RTW (Figure 1). Table 3 shows that the self-employed were 70% more likely to RTW at one month than employed participants (RR 1.70, 95% CI 1.17 to 2.47) and those with a moderate or severe injury were 52% less likely to RTW than those with a minor injury (RR 0.48, 95% CI 0.32 to 0.72).

**Table 3:** Factors associated with full RTW at 1 month post injury for emergency department attenders, frequencies (row percentage) and unadjusted and adjusted relative risks

	**RTW (N=71) n (%)**	**Not RTW (N=124) n (%)**	**Unadjusted relative risk (95% CI)**		**Model 1 Adjusted**^1^**relative risks (95% CI)**		**Model 2 Adjusted**^1^**relative risks (95% CI)**	
Study centre								
Nottingham	16 (34.8)	30 (65.2)	1		1		1	
Bristol	20 (42.6)	27 (57.4)	1.22	(0.73 to 2.05)	1.27	(0.76 to 2.13)	1.15	(0.71 to 1.85)
Swansea	21 (31.3)	46 (68.7)	0.9	(0.53 to 1.53)	1.14	(0.66 to 1.97)	1.07	(0.62 to 1.82)
Surrey	14 (40.0)	21 (60.0)	1.15	(0.65 to 2.03)	1.2	(0.66 to 2.18)	1.09	(0.62 to 1.91)
Sex								
Females	33 (34.0)	64 (66.0)	1		1			
Males	38 (38.8)	60 (61.2)	1.14	(0.78 to 1.66)	1.05	(0.71 to 1.57)		
Age								
16-35 years	29 (36.3)	51 (63.8)	1		1			
36-55 years	28 (35.4)	51 (64.6)	0.98	(0.64 to 1.48)	0.99	(0.62 to 1.56)		
56-65 years	14 (38.9)	22 (61.1)	1.07	(0.65 to 1.77)	0.9	(0.50 to 1.62)		
Ethnicity								
White UK	69 (37.7)	114 (62.3)	1		1			
Other	2 (16.7)	10 (83.3)	0.44	(0.12 to 1.59)	0.42	(0.14 to 1.28)		
Deprivation (Townsend score tertiles)								
1 (least deprived)	32 (37.2)	54 (62.8)	1		1			
2	22 (34.4)	42 (65.6)	0.92	(0.60 to 1.43)	0.95	(0.64 to 1.41)		
3 (most deprived)	17 (37.8)	28 (62.2)	1.02	(0.64 to 1.62)	1.13	(0.69 to 1.85)		
Employment								
Paid employment	56 (32.9)	114 (67.1)	1		1		1	
Self-employed	15 (60.0)	10 (40.0)	1.82	(1.24 to 2.68)	1.69	(1.03 to 2.76)	1.7	(1.17 to 2.47)
Long-term illness								
No	65 (36.1)	115 (63.9)	1		1			
Yes	6 (40.0)	9 (60.0)	1.11	(0.58 to 2.12)	0.94	(0.50 to 1.78)		
Place of injury								
Home	21 (44.7)	26 (55.3)	1		1			
Work	8 (27.6)	21 (72.4)	0.62	(0.32 to 1.21)	0.55	(0.29 to 1.06)		
Road	13 (32.5)	27 (67.5)	0.73	(0.42 to 1.26)	0.81	(0.44 to 1.49)		
Sports grounds	9 (39.1)	14 (60.9)	0.88	(0.48 to 1.60)	1.13	(0.54 to 2.35)		
Other	20 (35.7)	36 (64.3)	0.8	(0.50 to 1.29)	1.08	(0.63 to 1.86)		
Type of injury								
Other	36 (49.3)	37 (50.7)	1		1			
Upper limb injury	16 (27.6)	42 (72.4)	0.56	(0.35 to 0.90)	0.69	(0.40 to 1.18)		
Lower limb injury	19 (29.7)	45 (70.3)	0.6	(0.39 to 0.94)	0.77	(0.46 to 1.30)		
Severity								
Minor	48 (50.0)	48 (50.0)	1		1		1	
Moderate to severe	23 (23.2)	76 (76.8)	0.46	(0.31 to 0.70)	0.52	(0.32 to 0.85)	0.48	(0.32 to 0.72)
Injury intent								
Unintentional	68 (36.2)	120 (63.8)	1		1			
Other	3 (42.9)	4 (57.1)	1.18	(0.49 to 2.85)	1.22	(0.46 to 3.21)		

^1^ all variables in the column were included into the model.

At four months, 83% (159/191) of ED attenders had fully RTW (Figure 1). Table 4 shows that injury severity was significantly associated with RTW, with those with a moderate or severe injury being 21% less likely to RTW than those with a minor injury (RR 0.79, 95%CI 0.68 to 0.92). The self-employed were 15% more likely to RTW than employed participants (RR 1.15, 95%CI 1.03 to 1.30). Analyses were robust to the sensitivity analyses described above.

**Table 4:** Factors associated with full RTW at 4 months post injury for emergency department attenders, frequencies (row percentage) and unadjusted and adjusted relative risks

	**RTW (N=140)**	**Not RTW (N=31)**	**Unadjusted relative risk (95% CI)**		**Model 1 Adjusted**^1^** relative risks (95% CI)**		**Model 2 Adjusted**^1^**relative risks (95% CI)**	
Study centre								
Nottingham	35 (83.3)	7 (16.7)	1		1		1	
Bristol	41 (87.2)	6 (12.8)	1.05	(0.88 to 1.25)	1.06	(0.88 to 1.28)	1.03	(0.87 to 1.22)
Swansea	35 (70.0)	15 (30.0)	0.84	(0.67 to 1.05)	0.92	(0.74 to 1.14)	0.87	(0.70 to 1.08)
Surrey	29 (90.6)	3 (9.4)	1.09	(0.91 to 1.30)	1.09	(0.90 to 1.33)	1.08	(0.91 to 1.28)
Sex								
Females	72 (81.8)	16 (18.2)	1		1			
Males	68 (81.9)	15 (18.1)	1	(0.87 to 1.15)	0.93	(0.80 to 1.08)		
Age								
16-35years	61 (85.9)	10 (14.1)	1		1			
36-55years	54 (80.6)	13 (19.4)	0.94	(0.81 to 1.09)	0.9	(0.76 to 1.06)		
56-65years	25 (75.8)	8 (24.2)	0.88	(0.71 to 1.09)	0.75	(0.59 to 0.97)		
Ethnicity								
White UK	132 (82.5)	28 (17.5)	1		1			
Other	8 (72.7)	3 (27.3)	0.88	(0.61 to 1.28)	0.84	(0.59 to 1.21)		
Deprivation (Townsend score tertiles)								
1 (least deprived)	58 (82.9)	12 (17.1)	1		1			
2	49 (79.0)	13 (21.0)	0.95	(0.81 to 1.13)	0.98	(0.84 to 1.14)		
3 (most deprived)	33 (84.6)	6 (15.4)	1.02	(0.86 to 1.21)	1.04	(0.86 to 1.27)		
Employment								
Paid employment	118 (79.7)	30 (20.3)	1		1			
Self-employed	22 (95.7)	1 (4.3)	1.2	(1.06 to 1.35)	1.29	(1.10 to 1.52)	1.15	(1.03 to 1.30)
Long-term illness								
No	126 (80.8)	30 (19.2)	1		1			
Yes	14 (93.3)	1 (6.7)	1.16	(0.99 to 1.35)	1.21	(0.98 to 1.49)		
Place of injury								
Home	36 (85.7)	6 (14.3)	1		1			
Work	24 (85.7)	4 (14.3)	1	(0.82 to 1.22)	1.01	(0.83 to 1.22)		
Road	19 (73.1)	7 (26.9)	0.85	(0.65 to 1.11)	0.86	(0.67 to 1.10)		
Sports grounds	19 (79.2)	5 (20.8)	0.92	(0.73 to 1.17)	0.98	(0.78 to 1.24)		
Other	42 (82.4)	9 (17.6)	0.96	(0.80 to 1.15)	1.1	(0.91 to 1.34)		
Type of injury								
Other	60 (88.2)	8 (11.8)	1		1			
Upper limb injury	40 (81.6)	9 (18.4)	0.93	(0.79 to 1.08)	1.11	(0.91 to 1.35)		
Lower limb injury	40 (74.1)	14 (25.9)	0.84	(0.70 to 1.01)	0.99	(0.80 to 1.24)		
Severity								
Minor	83 (91.2)	8 (8.8)	1		1		1	
Moderate to severe	57 (71.3)	23 (28.8)	0.78	(0.67 to 0.91)	0.75	(0.62 to 0.92)	0.79	(0.68 to 0.92)
Injury intent								
Unintentional	133 (81.6)	30 (18.4)	1		1			
Other	7 (87.5)	1 (12.5)	1.07	(0.82 to 1.41)	1.12	(0.82 to 1.52)		

^1^ all variables in the column were included into the model.

### Hospital admissions and work status following injury

One month after injury only 7% (10/146) of hospital admissions had fully RTW (Figure 1). At four months 57% (70/123) had fully RTW. Table 5 shows that men were almost twice as likely to have RTW at four months than women (RR 1.94, 95% CI 1.34 to 2.82) and those injured at work were 51% less likely to have RTW (RR 0.49, 95% CI 0.27 to 0.87) than those injured at home. Participants from more deprived areas were about 40% less likely to have RTW than those from the most affluent areas (participants from the most deprived tertile RR 0.59, 95% CI 0.40 to 0.85 and from the middle tertile RR 0.61, 95% CI 0.40 to 0.93 compared with the least deprived tertile). Analyses were robust to the sensitivity analyses described above.

**Table 5:** Factors associated with full RTW at 4 months post injury for hospital admissions, frequencies (row percentage) and unadjusted and adjusted relative risks

	**RTW (N=64)**	**Not RTW (N=50)**	**Unadjusted relative risk (95% CI)**		**Model 1 Adjusted**^1^**relative risks (95% CI)**		**Model 2 Adjusted**^1^**relative risks (95% CI)**	
Study centre								
Nottingham	18 (56.3)	14 (43.8)	1		1		1	
Bristol	12 (63.2)	7 (36.8)	1.12	(0.71 to 1.78)	1.28	(0.75 to 2.16)	1.13	(0.73 to 1.76)
Swansea	24 (48.0)	26 (52.0)	0.85	(0.56 to 1.30)	0.91	(0.58 to 1.41)	0.9	(0.58 to 1.39)
Surrey	10 (76.9)	3 (23.1)	1.37	(0.89 to 2.10)	1.24	(0.79 to 1.96)	1.29	(0.82 to 2.05)
Sex								
Females	20 (47.6)	22 (52.4)	1		1		1	
Males	44 (61.1)	28 (38.9)	1.28	(0.89 to 1.86)	1.65	(1.05 to 2.61)	1.94	(1.34 to 2.82)
Age								
16-35 years	15 (57.7)	11 (42.3)	1		1			
36-55 years	35 (61.4)	22 (38.6)	1.06	(0.72 to 1.57)	1.09	(0.75 to 1.59)		
56-65 years	14 (45.2)	17 (54.8)	0.78	(0.47 to 1.30)	0.83	(0.51 to 1.34)		
Ethnicity								
White UK	60 (55.6)	48 (44.4)	1		1			
Other	4 (66.7)	2 (33.3)	1.2	(0.66 to 2.17)	1.16	(0.62 to 2.14)		
Deprivation (Townsend score tertiles)								
1 (least deprived)	30 (69.8)	13 (30.2)	1		1		1	
2	17 (47.2)	19 (52.8)	0.68	(0.45 to 1.01)	0.56	(0.36 to 0.88)	0.61	(0.40 to 0.93)
3 (most deprived)	17 (48.6)	18 (51.4)	0.7	(0.47 to 1.03)	0.52	(0.34 to 0.78)	0.59	(0.40 to 0.85)
Employment								
Paid employment	47 (52.8)	42 (47.2)	1		1			
Self-employed	17 (68.0)	8 (32.0)	1.29	(0.92 to 1.80)	1.01	(0.67 to 1.52)		
Long-term illness								
No	58 (54.7)	48 (45.3)	1		1			
Yes	6 (75.0)	2 (25.0)	1.37	(0.88 to 2.12)	0.79	(0.41 to 1.52)		
Place of injury								
Home	18 (58.1)	13 (41.9)	1		1		1	
Work	8 (33.3)	16 (66.7)	0.57	(0.30 to 1.09)	0.5	(0.28 to 0.90)	0.49	(0.27 to 0.87)
Road	17 (65.4)	9 (34.6)	1.13	(0.75 to 1.70)	0.81	(0.52 to 1.27)	0.87	(0.57 to 1.32)
Sports ground	7 (50.0)	7 (50.0)	0.86	(0.47 to 1.58)	0.75	(0.38 to 1.46)	0.68	(0.37 to 1.23)
Other	14 (73.7)	5 (26.3)	1.27	(0.85 to 1.90)	1.32	(0.84 to 2.08)	1.23	(0.82 to 1.83)
Type of injury								
Other	32 (66.7)	16 (33.3)	1		1			
Upper limb injury	12 (63.2)	7 (36.8)	0.95	(0.64 to 1.41)	0.97	(0.65 to 1.45)		
Lower limb injury	20 (42.6)	27 (57.4)	0.64	(0.43 to 0.94)	0.6	(0.38 to 0.95)		
Severity								
Minor	17 (65.4)	9 (34.6)	1		1			
Moderate to severe	47 (53.4)	41 (46.6)	0.82	(0.58 to 1.15)	1.04	(0.68 to 1.59)		
Injury intent								
Unintentional	58 (55.8)	46 (44.2)	1		1			
Other	6 (60.0)	4 (40.0)	1.08	(0.63 to 1.84)	0.77	(0.42 to 1.41)		

^1^ all variables in the column were included into the model ^2^ P-value for exclusion from the model.

### Health status

Table 6 shows median EQ5D index and VAS scores at baseline, one and four months for ED attenders and for hospital admissions. The EQ5D index and VAS scores were significantly lower than baseline values for ED attenders and hospital admissions at both one and four months (all p values <0.001).

**Table 6:** Health status at baseline, one and four months post injury for ED attenders and hospital admissions, comparing one and four month values with baseline values

	**Baseline**	**One month**	**Comparison between baseline and one month**	**Four months**	**Comparison between baseline and four months**
	**Median (IQR)**	**Median (IQR)**	**P-value**^1^	**Median (IQR)**	**P-value**^1^
Attended ED					
EQ5D index	1 (1 to 1)	0.76 (0.66 to 1)	<0.001	0.80 (0.76 to 1)	<0.001
VAS score	90 (80 to 97)	80 (70 to 92)	<0.001	85 (74 to 95)	<0.001
Hospital Admission					
EQ5D index	1 (1 to 1)	0.59 (0.26 to 0.76)	<0.001	0.73 (0.62 to 0.80)	<0.001
VAS score	90 (80 to 99)	70 (55 to 80)	<0.001	80 (61 to 90)	<0.001

^1^ Wilcoxon test.

[ ]missing values.

Table 7 shows that the EQ5D index was significantly lower at one and four months post injury amongst ED attenders that had not fully RTW (1 month post injury p<0.001; 4 months post injury p=0.039) and hospital admissions (1 month post injury p=0.022; 4 months post injury p=0.003). The VAS score was only significantly lower amongst ED attenders that had not RTW at one month post injury (p=0.002).

**Table 7:** Health status at baseline, one and four months post injury for ED attenders and hospital admissions comparing those who had and had not fully RTW

	**RTW at one month**	**Not RTW at one month**	**Comparison by RTW status at one month**	**RTW at four months**	**Not RTW at four months**	**Comparison by RTW status at four months**
	Median (IQR)	Median (IQR)	P-value^1^	Median (IQR)	Median (IQR)	P-value^1^
Attended ED						
EQ5D index	0.80 (0.76-1.00)	0.69 (0.59-0.80)	<0.001	0.80 (0.80-1.00)	0.80 (0.69-1.00)	0.039
VAS score	80 (67–90)	90 (80–93)	0.002	85 (70–92)	86 (75–95)	0.202
Hospital Admission						
EQ5D index	0.76 (0.59-1.00)	0.52 (0.26-0.72)	0.022	0.78 (0.69-0.92)	0.69 (0.59-0.76)	0.003
VAS score	85 (69–90)	70 (55–80)	0.139	83 (70–90)	80 (60–90)	0.196

^1^Mann Whitney U test.

[ ]missing values.

**Figure 3:**
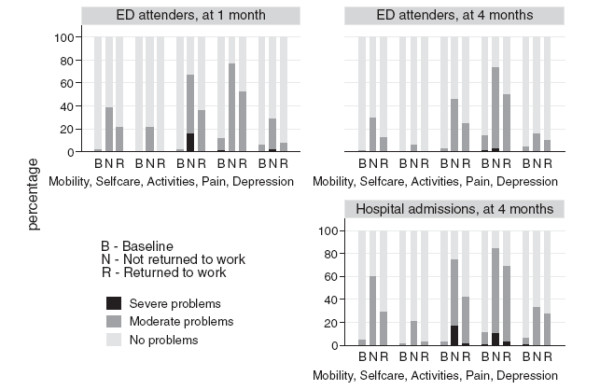
Health status (EQ5D index) for emergency department attenders at one month and four months and for hospital admissions at four months by full RTW.

Pain was the most common problem at all time points (Figure 3). Of those who had not fully RTW, 71% of ED attenders and 84% of hospital admissions had problematic pain four months after injury. Problems with usual activities and mobility were the second and third most commonly reported problems at all time points. At one month ED attenders who had not RTW reported significantly more problems in each domain than those who had (all p values <0.05). At four months, ED attenders who had not RTW reported significantly more problems with mobility and usual activities than those who had (both p values <0.05). At four months hospital admissions who had not RTW reported significantly more problems with usual activities, mobility and self care than those who had (all p values <0.05). One fifth of ED attenders reported anxiety or depression at 1 month and 7% at 4 months, whilst 27% of hospital admissions reported anxiety or depression at four months.

## Discussion

### Summary of main findings

Injuries have a large impact on health care use and time off work in the UK. This applies at both one and four months post injury, and even injuries that “only” required ED attendance have considerable impact four months post injury. Prognostic factors for RTW vary over time and between those attending ED and those admitted to hospital. Amongst ED attenders, injury severity and employment type (self employed or employed) predict RTW in the short term and medium term (four months). Amongst those admitted, gender, deprivation and place of injury predict RTW in the medium term. Health status was significantly poorer at one and four months after injury than before the injury and was significantly poorer amongst those that had not RTW compared to those that had. Many people experienced problems with pain, undertaking usual activities and mobility in the short and medium term, with a smaller proportion experiencing problems with anxiety and depression. Those who had not RTW experienced more problems than those who had, particularly with mobility and usual care activities in the medium term. A high proportion of injured people experienced problems with pain at all time points whether they had RTW or not.

### Strengths and limitations of the study

This is the largest UK study quantifying health care use and work impact resulting from a wide range of injuries. Our response rates are similar to one comparable UK study, [10] but lower than those from a UK study focussing on road traffic injury in ED attenders [25]. The differences we observed in characteristics between responders and non-responders may influence our estimates of rates of RTW in either direction. Firstly women and those with more severe injuries were more likely to respond at one and four months, and both these factors have been found to be associated with lower rates of RTW [8] suggesting our RTW rates may be underestimated. Secondly those with workplace injuries, [10] blue collar workers and those with lower educational levels have been found to have lower rates of RTW, [8] and our finding of lower response rates amongst those with workplace injuries and the more disadvantaged may indicate our RTW rates are overestimated. The same review found conflicting evidence about the relationship between age and RTW, [8] making it difficult to estimate the likely effect of differential response rate by age in this study. As we did not collect detailed occupational data, we could not explore how specific injuries affect RTW in different occupations. Previous studies demonstrate RTW may be less likely in more physically demanding jobs, [26] which may at least partly explain the lower rates of RTW with increasing deprivation in this study.

We did not send further questionnaires to those who reported being fully recovered at the previous follow up time point. We assumed that these people remained fully RTW at subsequent time points. This is likely to overestimate our RTW rates as some people will have further time off work because of their injury. Our study did not collect data on psychological or social functioning which may play an important role in RTW post injury [27,28]. Such factors may confound the relationship between prognostic factors and RTW that we found, or act as effect modifiers or mediators. Despite this being the largest UK study of the longer term effects of a range of injuries, small numbers, particularly in the four month analysis for hospital admissions may mean some negative findings may result from insufficient power. In addition, small numbers within injury categories prevented us from exploring the impact of specific injuries on RTW.

The study did not collect data on benefits received by participants, hence the relationship between the benefits received and other factors associated with RTW could not be explored. The UK benefit system for those unable to work due to injury or illness is complex. Occupational sick pay entitlement schemes vary between companies with a minimum amount of statutory sick pay being paid to those whose companies do not provide their own sick pay schemes. At the time of the study, incapacity benefit was available to those unable to claim statutory sick pay (e.g. the self employed or unemployed). Those injured at work may have been eligible for Industrial Injuries disablement Benefit which is claimable 90 days or more following an accident at work and the sum payable is based on the degree of disability [29].

### Comparisons with existing literature

There are no directly comparable prospective UK studies with which to compare our findings. One study of employed men admitted to hospital in Sheffield in 1996 found 26% of participants had RTW 6 weeks post injury and 54% by six months post injury [10]. A study of consecutive road traffic injured ED attenders in Oxford found 69% were working by 3 months and 74% by one year [25]. We found only 7% of hospital admissions had RTW at 1 month and 57% at four months post injury. Despite differences in study populations and data collection time frames, our findings are broadly similar to these studies.

Our findings are consistent with others who found that factors predicting RTW vary over time [10,27,30,31] and that non-clinical factors (e.g. gender, deprivation, place of injury) are often more important than clinical factors (e.g. injury type or severity) in predicting RTW [8-10,22,26,27,31,32]. Potential explanations for gender and deprivation differences in RTW may include differences in the nature of work undertaken, in rates of psychological morbidity post injury and in physical and psychosocial outcomes post injury [8,22,26,27,32-38]. Our findings are also consistent with research suggesting that those who had not RTW in the short or medium term post injury had poorer mental health, increased physical disability, pain and greater problems with social functioning [10]. Several studies suggest those experiencing a greater degree of pain are less likely to RTW [26,36]. Recent work suggests attitudes towards pain may also be important, with those believing they should not work with their current level of pain being significantly less likely to RTW [27].

### Implications for practice and research

If the recommendations of the recent review of sickness absence are implemented, the majority of those suffering injuries will require an in-depth assessment of their fitness to work, as most will have more than 4 weeks sickness absence. Fitness for work assessors can use the risk factors we found to identify those at risk of a later RTW and use this information to provide evidence-based interventions to help this group make a successful RTW.

Consultations with patients requesting “fit-notes” are valuable opportunities to identify and address on-going problems. Removing the need for GPs to provide “fit-notes” may discourage people from consulting and opportunities to maximise recovery may be missed. Whilst in-depth assessments of fitness to work may help people RTW more quickly, recovery is a broader concept than just RTW. It is therefore important that, as part of the assessment process, people are encouraged to consult GPs with any on-going physical and psychological problems. Such consultations are likely to need to focus on improving pain control, exploring attitudes towards pain, addressing mobility problems, and identifying and managing anxiety and depression.

Further research is needed to provide a more comprehensive assessment of the complex relationships between physical, psychological, social and occupational outcomes for a wide range of injuries of varying mechanisms and severities in the UK. This includes exploring the determinants of persistent pain, mobility problems and psychological problems following injury. In order to investigate the impact of specific injury types on RTW considerably larger studies will be required. Such research is needed to inform the design and evaluation of potential interventions for maximising recovery and ensuring successful RTW. In addition, if the recent review of sickness absence is implemented, it will be important to measure the impact of this on patient outcomes post injury. Assessing access to primary care services will be a key question, as primary care is ideally placed to address the most common physical and psychological problems after injury.

## Competing interests

The authors declare that they have no competing interests.

## Authors’ contribution

DK was the PI for the Nottingham site, contributed to study design, statistical analysis plan, and interpretation of results and drafting of the paper. YV contributed to the analysis plan, undertook the analysis and contributed to the interpretation of the results and drafting of the paper. CC contributed to the study design, statistical analysis plan, and interpretation of the results and drafting of the paper. NC was the PI for the Surrey site and contributed to study design, interpretation of results, and drafting of the paper. RL was the CI for the UK Burden of Injuries Study and PI for the Swansea site, contributed to study design, interpretation of results, and drafting of the paper. ET was the PI for the Bristol site and contributed to study design, interpretation of results, and drafting of the paper. All authors read and approved the final manuscript.

## Funding body

This work is based on independent research commissioned and funded by the Policy Research Programme in the department of Health (reference number 0010009). The views expressed are not necessarily those of the department.

## Pre-publication history

The pre-publication history for this paper can be accessed here:

http://www.biomedcentral.com/1471-2458/12/584/prepub
